# Stem/Stromal Cells for Treatment of Kidney Injuries With Focus on Preclinical Models

**DOI:** 10.3389/fmed.2018.00179

**Published:** 2018-06-15

**Authors:** Adriana Torres Crigna, Cristina Daniele, Carolina Gamez, Sara Medina Balbuena, Diego O. Pastene, Daniela Nardozi, Cinzia Brenna, Benito Yard, Norbert Gretz, Karen Bieback

**Affiliations:** ^1^Medical Faculty Mannheim, Institute of Transfusion Medicine and Immunology, University of Heidelberg, German Red Cross Blood Service Baden-Württemberg-Hessen, Mannheim, Germany; ^2^Medical Faculty Mannheim, Medical Research Centre, University of Heidelberg, Mannheim, Germany; ^3^Department for Experimental Orthopaedics and Trauma Surgery, Medical Faculty Mannheim, Orthopaedic and Trauma Surgery Centre (OUZ), Heidelberg University, Mannheim, Germany; ^4^Department of Medicine (Nephrology/Endrocrinology/Rheumathology), University Medical Centre Mannheim, University of Heidelberg, Mannheim, Germany

**Keywords:** mesenchymal stromal cells, MSC therapy, renal injury, acute kidney injury, chronic kidney injury, diabetic nephropathy, polycystic kidney disease, kidney transplantation

## Abstract

Within the last years, the use of stem cells (embryonic, induced pluripotent stem cells, or hematopoietic stem cells), Progenitor cells (e.g., endothelial progenitor cells), and most intensely mesenchymal stromal cells (MSC) has emerged as a promising cell-based therapy for several diseases including nephropathy. For patients with end-stage renal disease (ESRD), dialysis or finally organ transplantation are the only therapeutic modalities available. Since ESRD is associated with a high healthcare expenditure, MSC therapy represents an innovative approach. In a variety of preclinical and clinical studies, MSC have shown to exert renoprotective properties, mediated mainly by paracrine effects, immunomodulation, regulation of inflammation, secretion of several trophic factors, and possibly differentiation to renal precursors. However, studies are highly diverse; thus, knowledge is still limited regarding the exact mode of action, source of MSC in comparison to other stem cell types, administration route and dose, tracking of cells and documentation of therapeutic efficacy by new imaging techniques and tissue visualization. The aim of this review is to provide a summary of published studies of stem cell therapy in acute and chronic kidney injury, diabetic nephropathy, polycystic kidney disease, and kidney transplantation. Preclinical studies with allogeneic or xenogeneic cell therapy were first addressed, followed by a summary of clinical trials carried out with autologous or allogeneic hMSC. Studies were analyzed with respect to source of cell type, mechanism of action etc.

## Introduction

Renal failure is the impaired kidney function, which may lead to a systemic homeostasis disruption. Evidences show that kidney is a target organ of several diseases (hypertension, anemia, dyslipidemia, etc.), and when its physiology is compromised, it might initiate or exacerbate other pathophysiological conditions such as cardiovascular disease (1). Chronic kidney injury (CKI) is a long-term disease characterized by a decrease of glomerular filtration rate and an increase of albuminuria. CKI is associated with a higher risk of cardiovascular morbility, poor life quality, and early death, positioning CKI as health burden worldwide (2). CKI is frequently asymptomatic, and the ESRD requires dialysis or kidney transplantation (KTx), which is highly expensive for health care systems (3). In this field, cellular therapy with stem or stromal cells could potentially contribute to a better outcome of nephropathy.

Embryonic stem cells (ESC) are pluripotent cells with unlimited proliferative lifespan, derived from the inner mass of blastocysts (4). Their differentiation potential toward all cell lineages makes them highly attractive for cell-therapy approaches. However, despite the wide use of ESC in experimental settings, its clinical use is a matter of debate. As an alternative, induced-pluripotent stem cells (iPSC) were developed as differentiated cells genetically reprogrammed to induce ESC-like state, but these cells exhibit high risk of develop tumorigenicity (5, 6).

Multipotent mesenchymal stromal cells (MSC) are mesoderm derived, fibroblast-like and shuttle-shape cells under *in vitro* conditions (7). MSC can be isolated from a variety of adult or fetal sources like bone marrow, adipose tissue (ASC), periosteum, synovium, dental pulp, fetal tissues, umbilical cord tissue (UC), umbilical cord blood (UCB), placenta, and amniotic fluids (8, 9). Based on the MSC differentiation potential, stromal support, immunomodulatory properties and secretion of trophic factors, MSC are currently exploited in a variety of clinical settings (10) for tissue regeneration, immunomodulation, or graft improvement. Nevertheless, some of the MSC properties could change over the expansion phase *in vitro*. For instance, when bone marrow-derived MSC (BM-MSC) are isolated from their native tissue to culture flasks, the cells change from mostly quiescent and rounded-, actively proliferating and spindle-, to senescent and larger flattened-shape. Process, that is associated to loss of multipotency and also to DNA damage response activation (11). However, it has also been reported genetic instability in early passages, probably because of the initial adaptation of cells from their native niche to culture conditions (12). This creates a concern about a potential tumorigenic effect after MSC implantation.

The first cue of using MSC in regenerative medicine arose when Friedenstein et al. demonstrated the osteogenic potential of soft connective tissue bone marrow (BM)-derived stromal cells to form fibroblastic colonies *in vitro* (13–15). Heterogeneity and discordances in terminology and criteria for stromal/stem cells also spread with the increase of research in the field. Therefore, the International Society for Cellular Therapy remarked the appropriate designation as “multipotent mesenchymal stromal cells” (16).

The minimal criteria to define the human derived-MSC (hMSC) are: (1) Plastic-adherence under standard culture conditions, (2) More than 95% of the cells population must be positive for those antigens absent in most of hematopoietic cells such as CD105, CD73, and CD90. Moreover, up to 2% of the population can express CD45 (leucocytes), CD34 (hematopoietic progenitor), CD14 or CD11b (monocytes and macrophages), CD79α or CD19 (B cells), and HLA-DR (antigen presenting cell and lymphocytes), and (3) Differentiation potential to adipocytes, chondrocytes, and osteoblasts (17).

After their systemic administration, MSC move to inflammation sites, injury, and tumors to mediate healing (18). However, it also known that after intravenous delivery (IV), most of the MSC can be trapped mainly in the lungs, spleen, and liver (19–22). This diminishes the number of cells capable of homing and engrafting in the target organ. Therefore, proper imaging techniques to track and detect the specific MSC location in tissues could help to the further understanding of homing. There are several studies assessing the therapeutic potential of MSC in kidney disorders including CKI, focal segmental glomerular sclerosis, diabetic nephropathy (DN), autoimmune disease, and KTx (9). The rationale of MSC application is to cure or limit kidney diseases by the trophic factors that decrease fibrosis, promote angiogenesis, inhibit apoptosis, attenuate adverse inflammatory events by their immunomodulatory potential, and contribute to renal tissue regeneration (18, 23, 24). MSC immunomodulation generates an immunotolerant environment and reduces immune response of effector cells as monocytes/macrophages, dendritic cells, T or B cells (25–28). Paracrine effects mediated by extracellular vesicles (EVs) and exosomes further contribute to improve kidney function (23, 24).

The aim of this review is to present the results of a systematic search and analysis of the literature concerning the use of stem and stromal cells in different kidney diseases, focusing on preclinical models such as cisplatin-induced acute kidney injury (AKI), AKI induced by ischemia/reperfusion (AKI I/R), CKI, polycystic kidney disease (PKD), DN and KTx.

**Figure 1:**
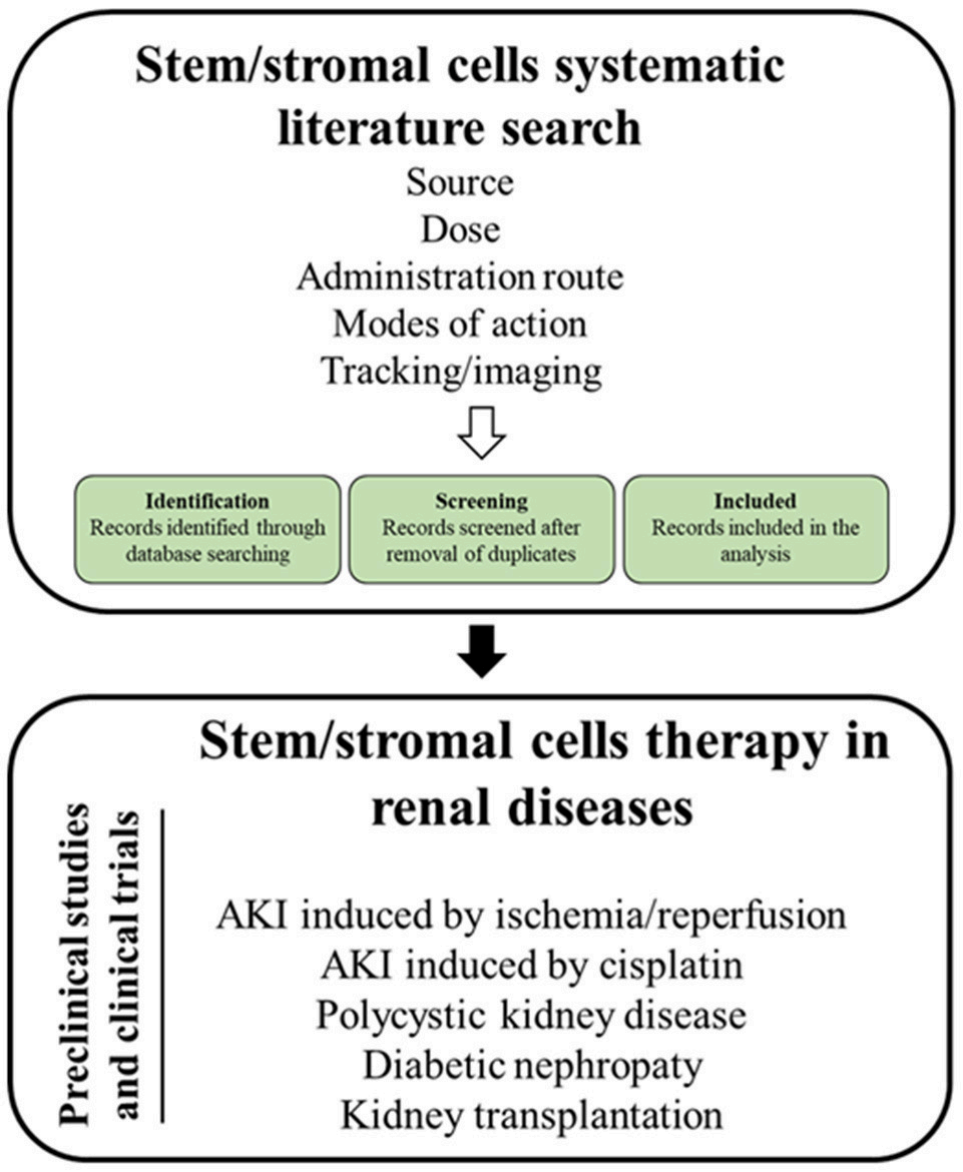
Graphical abstract.

As MSC are the most intensely studied cell type, the review will (1) summarize relevant MSC mechanisms of action, (2) sum up imaging modalities to track stem cells and to monitor therapeutic effects in the kidney, (3) introduce the applied search strategy, and (4) present the state of the art on MSCs therapy in the treatment of the aforementioned kidney diseases (Figure 1).

## MSC therapy in the treatment of kidney diseases: search strategy

### Search methods for identification of studies

We searched for all studies (up to August 2017) in which animal models and clinical trials of the aforementioned diseases were treated with stem cells (Table 1). No cell type was excluded from the search. Studies with both allogeneic and xenogeneic stem cell therapy were included. For the search, the following databases were used: PubMed, Web of Science Core Collection, Cochrane Library, CINAHL, Clinical Trial Gov, and WHO ICTRP.

**Table 1:** Search strategy used in the systematic reference research.

**Disease**	**Relevant terms**
AKI induced by chemotherapy	1: Stem cells AND
	2: Acute Kidney Injury (AKI) AND
	3: Cisplatin
AKI induced by ischemia/reperfusion	1: Stem cells AND
	2: Acute Kidney Injury (AKI) AND
	3: reperfusion Injury
Diabetic nephropathy	1: Stem cells AND
	2: Diabetic Nephropathy
Polycystic kidney disease	1: Stem cells AND
	2: Polycystic Kidney Disease
Kidney transplantation	1: Stem cells AND
	2: Kidney transplantation

### Selection and description of studies

**Figure 2:**
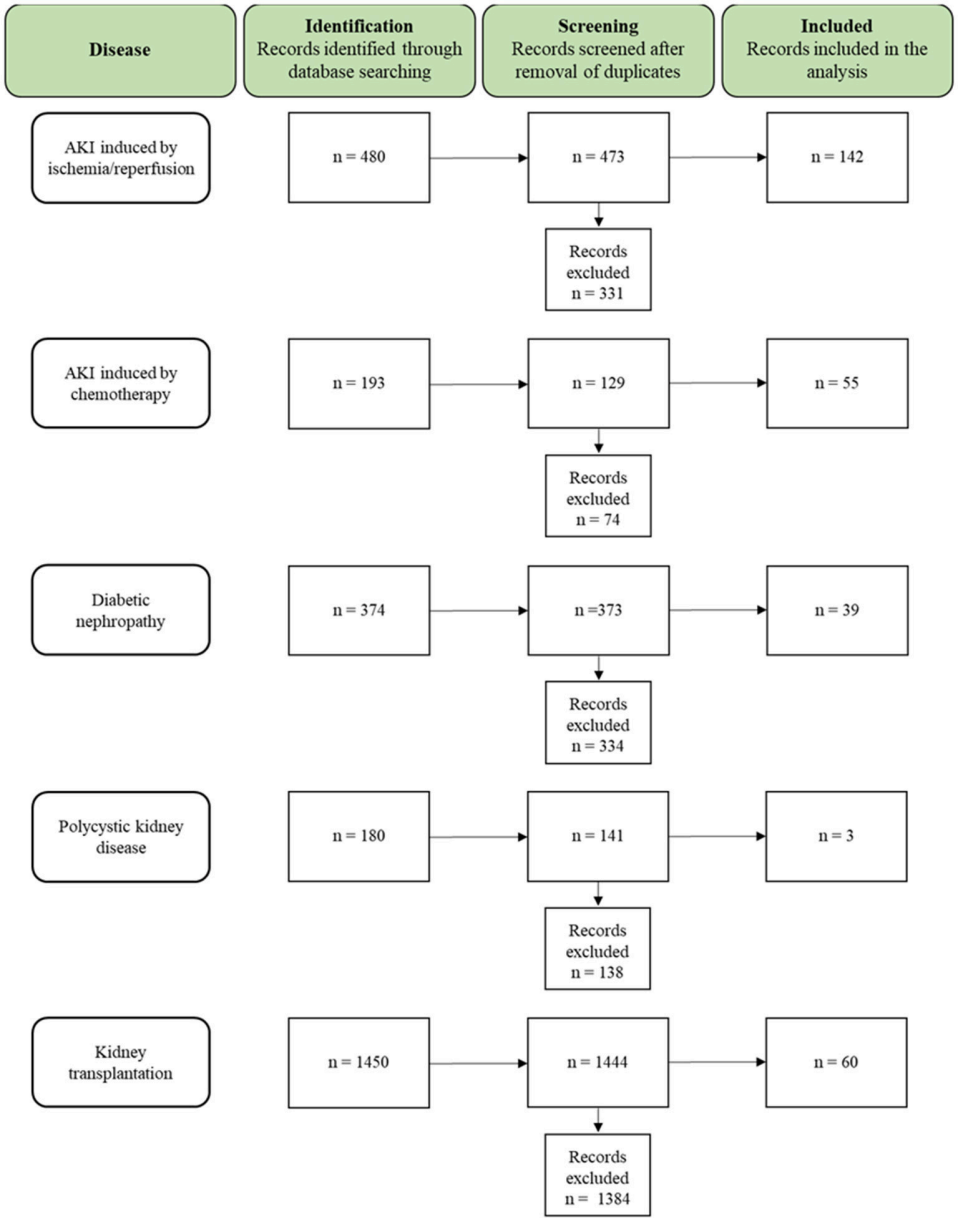
Flow chart summarizing the search strategy and the number of studies finally included or excluded from the analysis.

A total of 2,677 studies (AKI I/R *n* = 480; cisplatin induced AKI *n* = 193; DN *n* = 374; PKD *n* = 180; KTx *n* = 1450) were found through electronic searches by using the search terms (Figure 2).

As mentioned before, the aim of this review is to give an overview of the preclinical studies and clinical trials of the aforementioned kidney diseases performed so far in the stem cell therapy field.

According to this, the inclusion criteria were:

- preclinical studies with allogeneic and xenogeneic stem cells
- clinical trials with autologous and allogeneic stem cells

All the studies were reviewed in detail and, according to the inclusion criteria, 299 studies (AKI I/R *n* = 142; cisplatin induced AKI *n* = 55; DN *n* = 39; PKD *n* = 3; KTx *n* = 60) were identified as relevant for the study, while 2,378 studies (AKI I/R *n* = 331; cisplatin induced AKI *n* = 74; DN *n* = 334; PKD *n* = 138; KTx *n* = 1384) were excluded from the analysis.

## MSC modes of action

**Figure 3:**
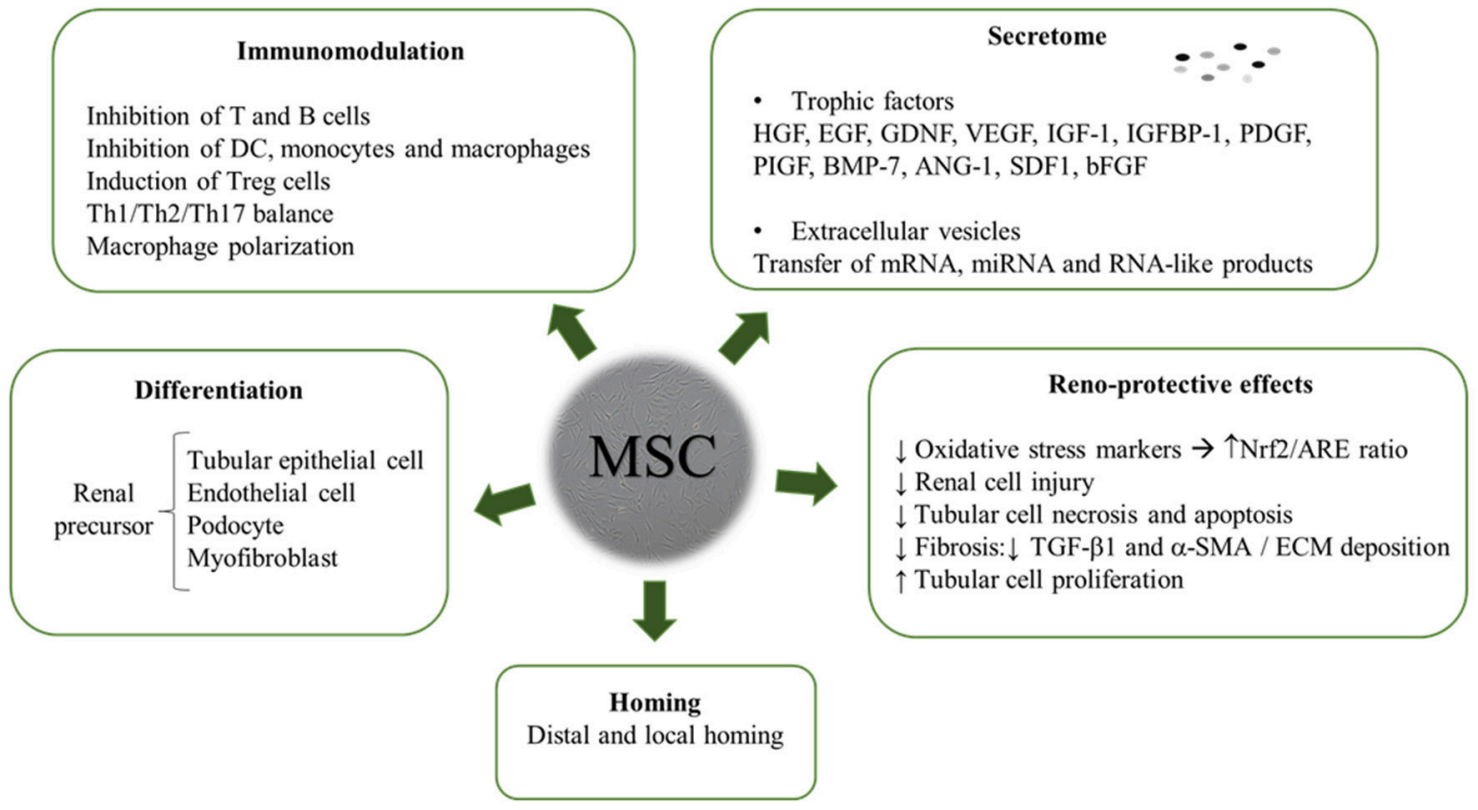
MSC modes of action in kidney environment.

The review yielded a high number of publications, which document therapeutic efficacy of stem cell-based therapies in kidney injury. MSC mainly exert their kidney renoprotection effects through paracrine and endocrine modes of action, that include immunomodulatory, anti-inflammatory, mitogenic, anti-apoptotic, anti-oxidative stress, anti-fibrotic, and angiogenic influences (Figure 3) (29–32).

### MSC immunomodulation

Immunomodulation emerges as key feature of MSC and refers not only to therapeutic mechanisms but also to their application as allogeneic cells. Despite expression of HLA/MHC class I and inducible expression of HLA/MHC class II, MSC present a hypo-immunogenic profile and appear to be transplantable across allogeneic/xenogeneic barriers (33). Conversely, in some studies with immunocompetent models administered with xenogeneic MSC, both humoral and cellular responses take place (34). Other allogeneic MSC animal studies suggest that a weak allogeneic immune response may be elicited (35).

Several studies claimed the lack of immune system activation after allogeneic/xenogeneic MSC administration (36). Also Le Blanc et al. suggest *in vitro* MSC immunosuppressive properties, but only a low number of studies support the translation in an *in vivo* setting (37).

On the other hand, both cellular and humoral immune responses took place *in vivo* after allogeneic MSC infusion, translating into a decreased engraftment (38, 39). In a study they speculate that MSC are rejected and this is a way to activate a regulatory immune response (40). Shuh et al. showed that alloantibodies were formed upon intravenous injection of allo-MSCs in rats, which facilitated complement-mediated lysis (41). Conversely, at least in the setting of graft-vs.-host-disease, immune-mediated killing of MSC was required to exert the therapeutic benefit (40).

Consequently, there is a need for further understanding on allogeneic/xenogeneic MSC infusion in the context of kidney injuries due to no consistent results and great discordances in both *in vivo* and *in vitro* settings. Some of the aspects to take into account when considering MSC treatment are MSC number of administrations, specific dosage, and interaction with immune suppression. Timing of MSC treatment is also of extreme importance given the interaction with the graft microenvironment (42). As this is a heavily debated feature, this review will specifically dissect studies using stem cell transplantation in allogeneic and xenogeneic settings.

Besides escaping immune recognition, MSC can act as immunomodulators by inhibiting lymphocyte activation and proliferation (43). MSC act on all cell types of the immune system: functional capacities of activated T cells are suppressed and a regulatory T cell (Treg) phenotype induced, monocytes are polarized to the anti-inflammatory M2 phenotype, dendritic cells maturation is impaired, likewise B and NK cell functions (44). It has been shown that M2 macrophages secrete a number of factors that mediate wound healing, sustain angiogenesis, extracellular matrix (ECM) deposition, and tissue remodeling (45).

In the setting of kidney transplantation there are several underlying mechanisms critically involved in the MSC-induced graft protection. MSC indoleamine 2,3-dioxygenase (IDO) secretion and Treg generation seem to prevent acute allograft rejection and induce renal allograft tolerance (46). These general features observed were also corroborated in murine animal models of kidney injury. For instance, MSC injected into the renal artery soon after reperfusion were able to sustain expression of the scatter factor system (i.e., hepatocyte growth factor, HGF) and modify cytokine network to a tolerogenic setting. MSC injection reduced IFNγ and IL-6 levels in serum, while increasing IL-10 anti-inflammatory cytokine suggesting that MSC reset the balance between T helper 1 and 2. Furthermore, MSC infusion induce Treg, which significantly prolong graft survival by Treg-dependent mechanisms and suppress the receptor tyrosine kinase in monocyte/macrophage cells, thus preventing monocyte infiltration and acute rejection. As a whole, these data suggest that MSC prevent rejection due to a shift of T cells toward an immune suppressive phenotype and the recruitment of the scatter factor system (47).

### MSC secretome and extracellular vesicles

In the large variety of disease entities where MSC have been investigated, it became clear that the main mechanism of action does not involve homing and engraftment of infused cells, but rather their trophic activities which motivated Caplan to call these cells “medicinal signaling cells” (48). A number of proteins such as enzymes, cytokines and growth factors, are secreted by MSC, including also EVs, microvesicles (MVs) or exosomes. These data raise the speculation of not using MSC for injection, but rather to use their conditioned media (CM) or even EVs as drugs (see below) (49). There are multiple factors that mediate paracrine effects and confer renoprotection acting on inflammation, apoptosis and oxidative stress, such as nitric oxide, IDO, TGF-α, TGF-β, prostaglandin E2, HGF, IL-6, IL-10, VEGF, bFGF. In several studies it is claimed that the expression of proinflammatory cytokines IL1β, TNFα, IFN-γ were reduced and anti-inflammatory TGF-α, bFGF, and Bcl-2 were highly upregulated in MSC treated kidneys (50–54).

Recently, studies with EVs (MVs and exosomes) suggest their mediation to restore ATP supply by mitochondria transfer to damaged cells. In fact, Bi et al. observed that MSC derived CM not only increased tubular cell survival due to an induced proliferation and migration of kidney-derived epithelial cells *in vitro* but also limited renal injury *in vivo* (55). EVs are known to transfer mRNA, miRNA, proteins and organelles that might promote important phenotypic changes. In a glycerol-induced AKI mouse model, BM-MVs effects were assessed. The authors speculate that the MVs mRNA secretion are the final effectors of these effects. In *in vivo* settings, EVs were seen to accelerate the recovery of the injury of mice, demonstrating that treatment with MVs have an equal efficacy than MSC on their functional and morphological recovery (56–58).

The first requirement for *in vitro* biological effects of EVs is the entrance into target cells. MSC-EVs possess several adhesion molecules that are typically expressed by the MSC they are originated from (α-1, α-4, and α-5 integrins and CD44). CD44 and α-1 integrin are involved in the EV internalization within renal tubular epithelial. *In vivo* evidences confirm the requirement of mechanisms for organ targeting and cell uptake to achieve EV regenerative effects. Multiple studies identified the transfer of extracellular RNA as the main molecular mechanisms for EV therapeutic effects. In this context, there is both *in vitro* and *in vivo* evidence of a transfer of specific mRNA and subsequent translation into proteins in renal tubular cells. Moreover, *in vitro* studies indicate that MSC-EVs mediated transfer of insulin growth factor 1 (IGF-1) receptor mRNA to renal tubular cells increased cell proliferation (59, 60).

So far, two studies regarding application of MSC-EVs to patients have been published. Nassar et al. investigated the therapeutic impact of MSC-EVs in patients with CKI and not only saw significant improvements of the kidney function but TGF-β and IL-10 levels were also broadly increased (61). In addition, Kordelas et al. administered MSC-EVs in an allogeneic setting, in which EV treatment favored the status of the patients immune cells (62).

### Renoprotective modes of action

Kidney fibrosis is a deposition of ECM in the renal parenchyma that leads to ESRD. ECM is normally degraded by matrix metalloproteinases (MMPs) but a disadjustment in the equilibrium between MMPs and tissue inhibitors of metalloproteinases (TIMPs), causes an accumulation of ECM and an inhibition of degradation and matrix restoration. MSC seem to have a repressive effect on the expression of TIMPs, which would lead to the resolution of fibrosis. (63–67).

Oxidative stress is produced due to an imbalance between the production of free radicals in the mitochondrial electron transport chain, and reduced anti-oxidant defenses (68). Reactive oxygen species (ROS) are also accumulated, which lead to an upregulation of TGFβ1 and ECM proteins in glomerular mesangial cells expression, triggering mesangial expansion (69). MSC may have an antioxidative effect, thus leading to a reduction of organ oxidative damage (70). MSC avert ROS accumulation due to antioxidant upregulation and scavenging, mediated by soluble factor secretion. MSC secretion of exosomes takes part in inhibiting ROS metabolism key enzyme depletion, consequently decreasing oxidative stress injury (68, 69).

MSC role in angiogenesis and vascular remodeling may comprise upregulation of pro-angiogenic and pro-survival factors. Among these, VEGF-a, IGF-1, and HGF, but also EVs may be involved (71, 72). MSC secretion of VEGF, HGF, IGF-1 together with TGF-β, stanniocalcin-1, GM-CSF, and FGF-2 seem to be involved in exerting anti-apoptotic effects (73). In MSC treated AKI mouse models, a decrease in pro-apoptotic (Bcl-xs) and an increase in anti-apoptotic molecules (Bcl-2 and Bcl-xl) have been reported (74). Anti/pro-apoptotic ratio balance is required for an enhanced renal recovery (75).

In some models of kidney disease, e.g., cisplatin-induced AKI, apoptosis of tubular cells is a key pathomechanism. MSC have been shown to provide anti-apoptotic, pro-regenerative cues, e.g., by inducing pro-regenerative/anti-apoptotic gene expression, or by transferring mRNA/miRNA to injured cells (70, 76).

### Homing and hemocompatibility

It is a matter of intense debate whether migration of the cells to the injured sites is necessary to exert their therapeutic actions. In diverse kidney disease models such as AKI I/R (77, 78), cisplatin-induced AKI (79, 80), KTx (81), CKI (82), atherosclerotic renal artery stenosis (83), and DN (84) no significant evidence of cell migration to the kidney was found despite a therapeutic effect was observed. Cells show preferential accumulation in lungs, suggesting capillary engraftment and degradation thereafter (85, 86). Moreover, in an AKI I/R model, cells were no longer observed 7 days after their administration, indicating that they were cleared without any engraftment (87). Contrarily, the use of hiPSC in cisplatin induced-AKI showed extensive engraftment into the kidney that led to restoration of renal function and structure (88). Additionally, in a rat model of steptozotocin (STZ)–induced diabetes, IV injected MSC were detected in the kidney, accompanied by a reduced expression of IL-1β, IL-6, and TNFα (89). Reasons for these discrepant data might be the wide variation of MSC type used, the different administration routes, the lack of consensus on most suitable doses, the broad tracking techniques available, and of course, the use of different disease models. This indicates that more sophisticated methods are required for cell tracking (see below), or that systematic searches as provided herein, identify aspects for harmonization/standardization.

When cells are systemically administered through i.v. injection most MSC tend to accumulate in lung, spleen or liver (filtering organs) (90). Most cells infused i.v. were found to get trapped in the lung as microemboli, being potentially dangerous (91–93). It has been suggested that MSC systemical administration home to organs such as the spleen, providing additional benefits, such as a reduction of infiltrating inflammatory cells (94). MSC local infusion to the injured sites will not only deliver a higher amount of MSC to the kidney, but has also suggested MSC role in enhancing proliferation of endogenous kidney stem cells (95, 96). Nonetheless, direct intra-arterial MSC infusion has been reported to favor obstruction of the capillaries, such as microvascular occlusions (97, 98). MSC direct injection does not appear to be more beneficial compared to their systemic infusion. Further studies should be carried out, focusing on the differences regarding routes of administration considering long-term safety.

Some recent studies addressed MSC effects on hemocompatibility/thrombosis/instant blood mediated reaction and indicate that MSCs expressing tissue factor can induce thrombosis and instant blood mediated reactions. Oeller et al. suggest that selecting tissue-factor deficient or low expressing cells may improve therapeutic outcomes (99). With respect to this, clinical trial protocols have already been modified to add anti-thrombotics such as heparin or hirudin (100–103).

Our own data suggest, that MSCs do not induce platelet activation and thereby thrombus formation, but rather actively inhibit platelet activation by a CD73 activity generating anti-thrombotic adenosine (Netsch et al., submitted).

### Differentiation

Another interesting subject regarding MSC therapy is whether they have the potential, once into the injured sites, to differentiate beyond the osteo-, chondro-, and adipogenic lineage. Most of the data available in this regard comes from *in vitro* studies being *in vivo* reports still scarce (104–106). Nevertheless, some studies have demonstrated a successful differentiation of MSC into kidney cells. Immunohistochemical and laser-scanning microscopy analyses revealed that BM-MSC from syngeneic GFP transgenic donors are able to differentiate into glomerular mesangial cells in C57BL/6 mice (107). Additionally, in a mouse model of AKI I/R, hASC have shown differentiation capability to renal tubular epithelial cells at early stages of injury (108). Conversely, Tögel et al. using the same model in rats, demonstrate that MSC injected via carotid artery did not differentiate toward tubular or endothelial cells, but still a therapeutic effect was found (74).

The highly contradictory data based on different models indicate that further investigation is needed to better elucidate if local communication with injured cells is required or whether the therapeutic effects observed are the result of trophic actions of MSC. Furthermore, our current understanding of cell behavior *in vivo* might be compromised due to limitations of imaging techniques. Therefore, improvement of existing and establishment of novel tracking and visualizing techniques is greatly needed.

## MSC tracking and imaging

A central question for MSC-based therapies is: where do cells go after their transplantation? One of the limiting factors of MSC transplantation is their interaction with the microenvironment (109). Therefore, it is essential to provide information about stem cells homing, motility, migration, and allocation (110). Accordingly, the use of the microscopy for both localization and quantification of stem cells within target tissues has increased and different methodologies have been developed (111).

For MSC tracking, direct labeling of viable cells with either contrast agents (super paramagnetic iron oxide SPIO, Gd, FLI) or fluorescent dyes have been used. However, the most pronounced disadvantage of direct labeling is the decrease in signal strength as the marker decays due to cellular division. Consequently, several indirect labeling techniques, that use genetic modification of cell-reporter genes, have been developed. Green fluorescent protein-based tags (GFP, S65T, EGFP, CFP, YFP) are mostly used (112–116). The indirect labeling is more sensitive but it requires genetic engineering tools that may compromise cellular function (117).

Depending on the sample to visualize, the correct choice of microscopy is utterly important (Table 2). Optical Imaging (OI) refers to those methods that use light in the ultraviolet/near-infrared range to investigate tissues and cellular and molecular structures in living organisms (128). Fluorescence, confocal, light-sheet microscopy, and bioluminescence imaging have been used (119–127, 129). Alternatively, magnetic resonance imaging (MRI) is vastly used and the resulting image is dependent on the type of contrast medium (110). Over the last thirty years, live cell imaging significantly improved with the development of incubation chambers, making it possible to analyze biological events in real time (130–132). *In toto* imaging is now commonly used to image and track cell movements and divisions (133).

**Table 2:** Description of the most commonly used microscopy for imaging.

**BrightField**	**Principle**	**Advantages**	**Drawbacks**	**References**
**WIDEFIELD MICROSCOPY**				
	Sample directly hit by the white light	Easy to use	Resolution around 200 nm	(118)
**OPTICAL IMAGING**				
Fluorescence Microscopy	Sample is illuminated by ultra-violet light to excite the fluorescent dye within the sample; Separation of emitted light from the excitation light (bright), by specific filters	Possibility to perform experiments under physiological conditions, making unnecessary chemical fixation and, therefore, minimizing artifacts	Risk of photobleaching and quenching related to the exposure time	(119, 120)
Bioluminescence Imaging	Detection of light emitted from cells, in which enzymes generating light are expressed, by genetic engineering. These enzymes belong to the luciferase group	High sensitivity; Cost- effectiveness; High reproducibility; Non-invasive	Use of genetic engineering tools (luc/neo-MSC); Low resolution; Low penetration depth; Low quantification accuracy; High light scattering	(121, 122)
Confocal Microscopy	Each spot scanned by the laser, back-and-forth	Imaging completed pixel by pixel; Use of the pinhole; Final image as an ensemble of layers, much detailed; Non invasive	Expensive; High sensitivity; Trained operators required	(119, 123, 124)
Two-Photon Microscopy	Use of two low-energy photons, usually from the same laser, cooperating to cause a higher-energy electronic transition in a fluorescent molecule (usually near-infrared light is used)	High resolution; Minimized scattering light; Increased penetration depth; Reduced photobleaching Reduced Background strongly; Increased total signal-to-noise ratio	Low penetration depth; Poor possibility of longitudinal studies	(125, 126)
LightSheet Microscopy	Use of a thin plane of light, instead of a point	Very fast imaging speed; Very high resolution; Very high penetration depth (>1 cm); Reduced photobleaching; Reduced phototoxicity	Dual side illumination not easy to be perfectly aligned in the three-dimensional space, causing a less sample focus, compared to the single side illumination	(127)
**MRI**	**Principle**	**Advantages**	**Drawbacks**	**References**
**MAGNETIC RESONANCE IMAGING**				
	Alignment of the magnetic moment from endogenous molecules (^1^H and ^19^F) into an external magnetic field	High resolution; High sensitivity of cell detection; High penetration depth;	Low possibility of longitudinal studies; High costs; Presence of external metal disturb the analysis	(110)

In the kidney setting, various different labels and visualization techniques have been applied (Table 3).

- Magnetic polymeric nanoparticles to label MSC, for visualizing them by fluorescence microscopy (109).
- MRI visualization in a mouse model with ASC labeled with TMADM-03 (trimethylamino dextran-coated magnetic iron oxide nanoparticle 03) (134).
- Bioluminescence imaging as sensitive and specific *in vivo* cell tracking of luciferase/neomycin phosphotransferase-marked MSC (luc/neo-MSC) (138).
- Labeling of hMSC-EVs with 1,1-Dioctadecyl-3,3,3,3-tetramethylindodicarbocyanine (DiD) visualized by OI to assess biodistribution and renal localization (135). DiD and OI visualization has also been used in other studies (136, 137).

**Table 3:** MSC studies using different labels and visualizing techniques.

**Cell type**	**WJ-MSC**	**ASCs**	**hMSC-EV**	**BM-hMSC**	**hMSC; hESC**	**MSC**
Label	MPNPs	TMADM-03	DiD	DiD	DiD, ICG	luc/neo-MSC
Species	NK	Mouse	Mouse	Rat	NK	Mouse
Administration route	NK	SC	IV	IP	NK	IA
Target organ	NK	Skin, kidney	Kidney	Arthritic joints	NK	Kidney
Technique	Fluorescent Microscopy	MRI	OI	OI	OI	Bioluminescence imaging
Reference	(109)	(134)	(135)	(136)	(137)	(138)

### Optical tissue clearing

Bioimaging techniques have been optimized in the past with the aim to enhance imaging resolution to the subcellular level and to use them for *in vivo* studies. The high scattering of turbid biological tissues limits the penetration of light, light scatters, as a result of mismatch of the different refractive indexes (RIs) the image blurs and contrast decreases (139). In order to overcome this limitation, optical tissue clearing (OTC) has been introduced already in 1911 (140). Substances used within OTC can reduce the scattering of tissues and make them transparent by reducing and/or removing the main sources of scattering light (e.g., lipids from the membrane bilayer) without compromising the structure integrity. Some examples for clearing procedures are: BABB (benzyl alcohol-benzyl benzoate) (141), CLARITY (clear lipid-exchanged acrylamide-hybridized rigid imaging/immunostaining/*in situ*-hybridization-compatible tissue hydrogel) (142–144), PACT (passive CLARITY technique) (145), SeeDB (See Deep Brain) (146) and, recently, an Ethyl Cinnamate protocol was published (147).

Each type of tissue clearing includes four main technical steps: (1) initial treatment to eliminate pigment molecules, which may absorb tissue autofluorescence and affect the results of clearing, (2) permeabilization to assist the appropriate diffusion of the clearing solution throughout a tissue specimen (mostly using Triton X-100, other detergents or DMSO, (3) immunolabeling with different fluorescent probes specifically or unspecifically labeling target proteins, and (4) refractive matching clearing. Typical OTC substances have high refractive indices and when they permeate into the tissue replace intersticial fluid or cytoplasm, which have a lower refractive index than the surrounding tissue. By this, differences in refractive indices are reduced and light can penetrate deeper. By combining the OTC with a suitable microscopy, significant improvements in 3D tissue visualization were achieved.

It is beyond the scope of this review to summarize the major achievements in OTC, which have been already reviewed (148, 149). Here we aim to highlight the recent developments for 3D whole organ imaging which is key for monitoring stem cell/tissue interaction. By choosing the optimal clearing technique and microscopy, it is possible to visualize, track and trace, and quantify stem cells within the whole organ in 3D.

## MSC therapy in the treatment of kidney diseases: state of the art

**Figure 4:**
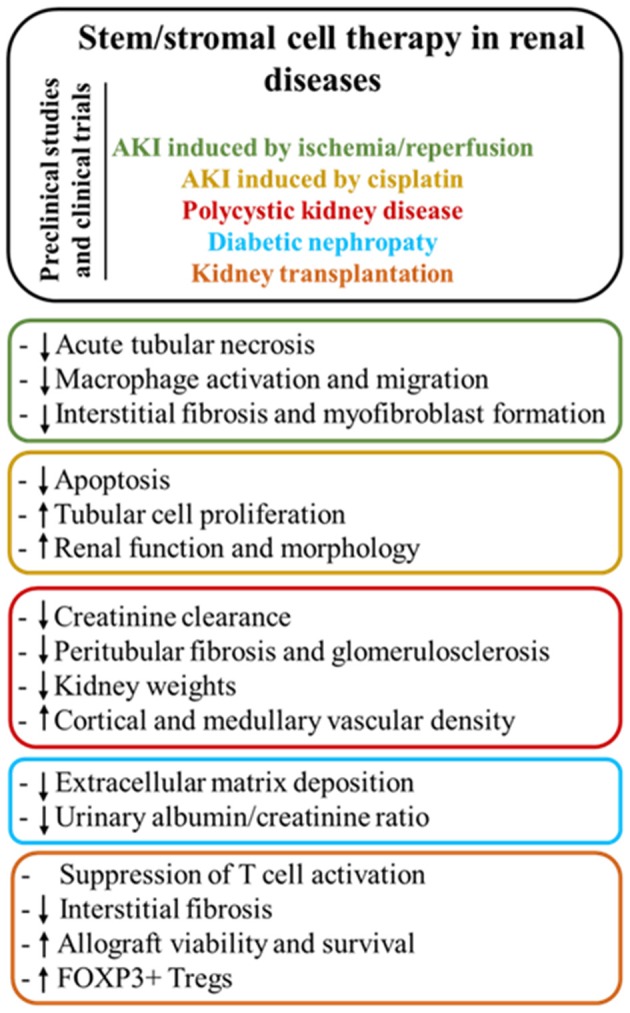
Main modes of action of stem/stromal cell therapy according to the different renal diseases.

The data from the systematic reference search were stratified first according to the different disease entities, where we will briefly introduce the respective models (Figure 4). Second, the most relevant preclinical studies with allogeneic or xenogeneic (Tables 4a–c) cell therapy were analyzed and discussed in order to give an overview of the mechanisms of action mainly involved in the renoprotective activity of MSC. Lastly, a summary of the clinical trials carried out with the use of autologous or allogeneic hMSC is provided (Table 5).

**Table 4a:** Xenogeneic MSC therapy in AKI I/R preclinical studies.

**Model**	**Animal/Strain**	**Treatment**	**Dose**	**Adm.route**	**Outcome**	**References**
AKI I/R	Rat SD	hASCs	2 × 10^6^ in 500 μl EBM-2/1% BSA	IA (abdominal aorta)	↓SCr, tubular damage ↓T cell infiltration in kidneys ↑Tregs	(150)
AKI I/R	Rat Wistar	hUC-MSCs hASCs	1 x 10^6^cells in 2 mL saline	IP	UC-MSCs more prominent renal function protection compared to ASCs treated. Amelioration of long-term renal function. ↓β-galactosidase and ↑ Klotho compared to ASCs	(151)
AKI I/R	Rat SD	hSVF hASCs	2 x 10^6^cells in 100 μl PBS	IR	↑Cell proliferation ↓Apoptosis, IL-10, TNF-α	(152)
AKI I/R	Mouse C57BL/6	hASCs		IV (tail vein)	↑ Proliferation, tubular sox9 by release of exosomes ↓TGF-β1	(153)
AKI I/R	Rat Wistar	VEGF-hAFSCs	1 x 10^6^cells or 5 x 10^5^cells	IA (aorta)	Treatment with higher dose: ↑ SCr, BUN, Acute tubular necrosis, hyaline cast formation. Treatment with lower dose: ↑ Proliferation, FOXP3+ cells.	(154)
AKI I/R	Rat Wistar	hUCB-MSC	1 x 10^6^cells	IV (tail vein)	↓ SCr, BUN, oxidative stress.	(155)
AKI I/R	Mouse SCID	h Gl-MSC hGl-MSC-EVs Progenitor cells from cortical tissue T-CD133+ cells T-CD133+ cells EVs	1 x 10^5^cells 400–480 x 10^6^Evs	IV (tail vein)	hGl-MS-EVs: more efficiently improved renal function compared to T-CD133+ EVs. hGl-MSCs: ↑ proliferation of tubular cells. RNAse treated EVs: ineffective	(156)
AKI I/R	Mouse C57 BL/6	hERCs	1 x 10^6^cells	IV (tail vein)	↓ SCr, BUN, TNF-α, IL-6, IFNγ, splenic and renal CD4+, CD8+ T cells. ↑CD4+, CD25+ Tregs and M2 macrophages	(157)
AKI I/R	Mouse FVB	Micro RNA-486-5p from hECFCs-derived exosomes	20 μg	IV (yugular vein)	↓SCr, BUN ↓Apoptosis ↓Neutrophil infiltration	(158)
AKI I/R	Rat	hWJ-MSC-EVs	100 μg EVs	IV (cava caudalis)	↓Apoptosis, sNgal ↑Nrf2/ARE, HO-1	(159)
AKI I/R	Rat	hUC-MSC	100 μg	IV (caudal vein)	↓Apoptosis ↑VEGF ↓HIF-1α	(160)
AKI I/R	Rat Sprague-Dawley	hWJ-MSC-EVs	100 μg	IV (caudal vein)	↓Cell apoptosis ↓Expression of miRNA-30	(161)
AKI I/R	Mouse NOD.CB1-Prkdc ^scid/J^	hUC-MSC and hUC-MSC-EVs	1 *x* 10^6^in 100 μl PBS	IV (yugular vein)	↓ SCr, tubular necrosis, oxidative stress, apoptosis. No cell persistence in kidneys.	(30)
AKI I/R	Rat SD	hUC-MSC-MVs	30 μg	IV (cava caudalis)	↓Collagen deposition, proliferation tubular cells. ↑HGF mRNA expression. TGF-β1, IGF-1 and EGF not affected. mRNAse treatment abolishes renoprotective effect	(162)
AKI I/R	Mouse C57BL/6	hWJ-EPCs	5 x 10^5^cells	IR (subcapsular space)	Cells found in cortex at 1–2 days post AKI and in medulla and cortex 7 days post AKI. ↓ SCr, tubular necrosis and dilation ↑Microvascular density	(163)
AKI I/R	Rat Wistar	hAFSCs with renal progenitor phenotype	1 x 10^6^ cells in 800 μl fresh expansion media	IA (intra aorta, directed to kidney by subsequent clamping)	↓ SCr, tubular necrosis, cast formation, macrophage infiltration, myofibroblast formation, interstitial fibrosis	(164)
AKI I/R	Mouse NOD.CB1-Prkdc ^scid/J^	hiPSCs	1.5 x 10^6^cells	IR (subcapsular)	↓ SCr, BUN, tubular necrosis, interstitial fibrosis	(165)
AKI I/R	Mouse C57BL/6	hSHEDs	1 x 10^6^cells in 10μl PBS	IR (subrenal capsule)	↓ SCr, BUN, infiltration of macrophages and neutrophils, MIP-2, IL-1β, MCP1	(166)
AKI I/R	Rat SD	hASCs hypoxia preconditioned	2 x 10^6^cellsin 100μl saline	IR (renal cortex)	↓ SCr, BUN, apoptosis, histological injury. ↑Vascularization In hypoxia preconditioned compared to non-hypoxia preconditioned	(78)
AKI I/R	Mouse C57BL/6	hBM-MSC	1 x 10^6^	IV	Homing to kidneys ↓SCr, BUN, KIM-1 M2 macrophage polarization	(167)
AKI I/R	Rat SD	hWJ-MSC-MVs	100 μg MVs	IV (cava caudalis)	↓Tubular necrosis, apoptosis, CD68+ macrophages, CX3CL1, α-SMA. ↑Cell proliferation, IL-10	(168)
AKI I/R	Mouse C57BL/6	hUCB-MSC	1 x 10^6^ before AKI	IP	↓IFN⋎ ↑VEGF ↑%Tregs	(169)
AKI I/R	Rat SD	hWJ-MSC	2 x 10^6^ in 500 μl serum-free medium	IV	Shifting HGF/TGF-β1 to HGF Reduction in overall kidney fibrosis	(170)
AKI I/R	Mouse C57BL/6	hUC-MSC	2 x 10^6^ 24 h after AKI	IV (caudal vein)	↓Apoptosis ↑M2 macrophages	(171)
AKI I/R	Rat SD	hUCB-MSC- EVs	EVs only EVs+IFN⋎	IA (carotid artery)	EVs only ↓serum creatinine, urea, cute tubular necrosis. EVs+ IFNγ No ameliorative effect	(172)
AKI I/R	Mouse NOD.CB1-Prkdc ^scid/J^ and FVB/NJ	hUCB-MSC CD133+	1 *x* 10^6^cells in 100 μl saline	IV (jugular vein)	↑ SCr and urea, k+ and PO4, tubular injury	(173)
AKI I/R	Rat Wistar	EPC-MVs	30 μg	IV	↑Tubular cell proliferation ↓Apoptosis, leukocyte infiltration, interstitial fibrosis. Loss or renoprotective effect when treated with RNAse, Dicer knock-down or depletion of miRNA-126 and miRNA-296	(174)
AKI I/R	Rat SD	hBM-MSC- MVs	30 μg	IV	↓ SCr, BUN in acute phase, apoptosis, kidney fibrosis. Treatment with RNAse abolish renoprotective effects	(175)
AKI I/R	Rat Wild type	hMSCs from fetal membranes	1 x 10^6^ in 150 μl PBS MSCs only or pre-treated with butyric acid	IR	↓ SCr, urea, IFNγ, IL-1β, IL-1α, IL-6. ↑VEGF	(176)
AKI I/R	Rat	Hepatocyte growth factor modified hUC-MSCs	Not specified	IA (carotid artery)	↓SCr, BUN ↓Hyperemia, tubular cast formation ↓Caspase-3 ↑Tubular cell proliferation	(177)
AKI I/R	Rat SD	hUCB-MSC	1 *x* 10^6^in 500 μl saline	IA (carotid artery)	No transdifferentiation into renal cells. ↓SCr, urea, caspase-3, IL-1β	(178)

**Table 4b:** Xenogeneic MSC therapy in Cisplatin-induced AKI preclinical studies.

**Model**	**Animal/Strain**	**Treatment**	**Dose**	**Adm. route**	**Outcome**	**References**
Cisplatin-induced AKI	Rat NIH-Foxn1^rn^	hKDCs	10^6^ cells in 500 μl PBS (twice)	IV (tail vein)	↓FITC-sinistrin t1/2, sCr, serum urea, urinary albumin, tubular luminal area	(86)
Cisplatin-induced AKI	Mouse C57BL/6	hUCB-MSC	10^6^ cells. Early and late treatment	IV (tail vein), IP	Time-sensitive effect of MSCs. hUCB-MSCs early treatment: ↓BUN, apoptosis, tubular injury scores. ↑Treg. ↑anti-inflammatory and ↓pro-inflammatory cytokines Renoprotective effect addressed to immunomodulation activity.	(31)
Cisplatin-induced AKI	Mouse BALB/cOlaHsd	hUC-MSC+ATG pre-treatment	5 x 10^5^ cells	IV	↓BUN, sCr, kidney weight, in situ inflammation and oxidative stress	(179)
Cisplatin-induced AKI	Rat SD	hASCs	5 x 10^6^ cells	IV (tail vein)	↓BUN, sCr, oxidative stress, histological indices of injury in the renal cortex and outer medulla.	(180)
Cisplatin-induced AKI	Rat SD	hASCs, hAFSCs	5 x 10^6^ cells	IV (tail vein)	↓BUN, sCr, oxidative stress. ↑Regeneration and proliferation achieved with hAFSCs compared to hASCs.	(181)
Cisplatin-induced AKI	Rat SD	hAFSCs	5 x 10^6^ cells	IV (tail vein)	↓BUN, sCr, oxidative stress, fibrosis. ↑Tissue regeneration. Renoprotective effect addressed to paracrine antioxidant activity.	(182)
Cisplatin-induced AKI	Mouse BALB/c nude	HIF-1α-hASCs	10^5^ cells per 200 μl	IV (tail vein)	↓BUN, sCr, TNF-α, tubular damage score. ↑Antiapoptotic activity, HO-1 gene expression	(183)
Cisplatin-induced AKI	Rat SD	hASCs	1–2 x 10^6^ cells in 1 ml saline	IV (tail vein)	↓BUN, sCr, apoptosis ↑Tubular cell proliferation	(184)
Cisplatin-induced AKI	Mouse NOD-SCID	RPC-hiPSCs	5 x 10^5^ cells	IV (tail vein)	↓ BUN, renal tubular damage.	(88)
Cisplatin-induced AKI	Mouse C3H	hBM-MSC + pFUS pre-treatment	10^6^ cells	IV (tail vein)	↓BUN, sCr, mouse TNF-α, apoptosis, necrosis. ↑Human IL-10, mouse VEGF. M1 to M2 macrophage phenotype shift. pFUS improves MSCs homing to injured kidneys and increases the aforementioned outcome.	(185)
Cisplatin-induced AKI	Rat SD	hASCs, hAFSCs	5 x 10^6^ cells	IV (tail vein)	↓sCr, tissue oxidative stress. ↑Tissue regeneration and proliferation. Renoprotective effect achieved by both cell types. An antioxidant activity is proposed.	(186)
Cisplatin-induced AKI	Rat White albino	hUCB-HSCs	3 x 10^6^ cells	IP	↓BUN, sCr, TNF-α, HGF, IGF-1, VEGF, p53.	(187)
Cisplatin-induced AKI	Rat SD	hUC-MSC	2 x 10^6^ Cells in 500 μl saline solution	IV (tail vein)	↓BUN, sCr, apoptosis, IL-1b, and TNF-α, inflammatory cell accumulation, kidney interstitial fibrosis. ↑Renal cell proliferation. Homing to the renal lesion site observed. EMT inhibition.	(32)
Cisplatin-induced AKI	Mouse BALB/c nude	hUSSC	10^5^ cells in 500 μl PBS	IV (tail vein)	No amelioration observed. No statistically significant changes in the levels of BUN, sCr, TGF-β 1, HGF, and IGF-1. Cell transplantation worsens the kidney architecture (↑ injury score)	(188)
Cisplatin-induced AKI	Rat SD	hUC-MSC-Exs, hUC-MSC- CM	Exs: 200 μg. CM: not specified	Renal capsule injection (both kidneys)	Exs: ↓Oxidative stress, tubuli apoptosis. ↑ Cell proliferation. No significant changes in the levels of BUN and sCr observed. CM: No notable changes observed.	(189)
Cisplatin-induced AKI	Mouse C57BL6/J	hESC-MPs	5 x 10^5^ cells	IV (tail vein)	BUN, sCr, apoptosis, pro-inflammatory cytokines. ↑Anti-inflammatory cytokines, tubular cell proliferation. Cells engraftment in the kidney.	(190)
Cisplatin-induced AKI	Rat SD	hADSC, hASC-CM	hASCs: 5 x 10^5^ cells, hASC-CM: 4 ml	hASCs: IV (tail vein), hASC-CM: IP	hASCs and CM: ↓BUN, sCr, renal tissue injury, tubular apoptosis, TNF-α, NF-kB, COX2. ↑Animal survival. However, additional studies are needed to clarify if the protective effects of CM are equivalent to Ad-MSC treatment.	(79)
Cisplatin-induced AKI	Rat SAS- SD	hBM-MSC-CM	1 ml	IV (penile vein)	↓BUN, sCr, renal tissue injury, tubular apoptosis, IL-1β, TNFα, IL-6 and IL-1ra. ↑IL-10, animal survival	(191)
Cisplatin-induced AKI	Mouse NOD-SCID	hAFSCs	5 x 10^5^ cells	IV	↓BUN, sCr, renal tissue injury, apoptosis. ↑Animal survival. Cells engraftment in the peritubular region. Preconditioning with GDNF enhances the regenerative potential of hAFSCs.	(192)
Cisplatin-induced AKI	Mouse SCID	hBM-MSC-MVs	Single dose: 10 μg. Multiple dose: 100 μg + 5 x 50 μg	IV (tail vein)	MVs single dose: ↓BUN, sCr. ↓↓Apoptosis. ↑Survival. MVs multiple doses: ↓↓BUN, sCr. ↓Apoptosis. ↑↑Survival. Kidney morphology restoration.	(56)
Cisplatin-induced AKI	Mouse BALB/c	hE-MSC, VEGF-hE-MSCs	5 x 10^5^ cells per 500 μl	IV (tail vein)	VEGF-hE-MSC can strengthen the renoprotective effect of MSCs by antiapoptotic effect and proliferation on peritubular capillaries.	(193)
Cisplatin-induced AKI	Mouse BALB/c	hUSSC-CM			No amelioration in terms of serum urea and creatinine, histopathologic examinations and physical activity score was found.	(194)
Cisplatin-induced AKI	Mouse NOD-SCID	hUCB-MSC	5 x 10^5^ cells	IV	↓BUN, renal tubular damage, oxidative stress, apoptosis, inflammation. ↑Animal survival, tubular cell proliferation, serum HGF, kidney mRNA HGF	(85)
Cisplatin-induced AKI	Mouse NOD-SCID	hBM-MSC	5 x 10^6^ cells	IP	↓BUN, amylase, phosphorous, alanine aminotransferase, creatinine, cytokines/chemokines (MIP-2, G-CSF, KC, IL, MCP-1, PDGF, TNF-α, GM-CSF, IL-6). ↑Animal survival.	(195)
Cisplatin-induced AKI	Mouse NOD-SCID	hBM-MSC	5 x 10^5^ cells(500 μl)	IV (tail vein)	↓BUN, sCr, renal tissue injury, tubular cell apoptosis, peritubular capillary changes. ↑Animal survival.	(196)

**Table 4c:** Xenogeneic MSC therapy in DN preclinical studies.

**Model**	**Animal/ Strain**	**Treatment**	**Dose**	**Adm. route**	**Outcome**	**References**
STZ–T1D	Rat SD	hUDSC-Exosomes	100 μg exosomes	IV (tail vein)	↓Mesangial expansion, ↓glomeruli hypertrophy ↓Casp-3, and, ↓U-ACR (↑BMP-7, ↑VEGF, ↑TGF-ß and ↑angiogenin related)	(197)
STZ–T1D	Rat SD	hUCB-MNCs	0.5 x10^6^ cells	IV (tail vein)	↓ EMT markers (↓α-SMA and ↑ E-cadherin) ↓ Fractional mesangial area in glomeruli	(198)
STZ–T1D	Rat Albino	hUCB-MNCs	150 x 10^6^ cells	IV (tail vein)	↓ECM deposition (↓laminin expression) ↓U-ACR, ↓Arterial pressure	(199)
STZ–T1D	Mouse NOD.CB17-*Prkdc^*scid*^*/J	hBM-MSC	2.5 x 10^6^ cells (twice)	IC	↓Blood glucose ↓Macrophages infiltration, ↓Mesangial ECM deposition ↑Insulin Engraftment detected in pancreatic and renal histology	(84)

### Acute and chronic kidney injury

AKI is a clinical syndrome characterized by rapid decline in glomerular filtration rate, resulting in retention of nitrogenous waste, mainly serum creatinine (sCr) and blood urea nitrogen (BUN) (219). It is a worldwide health concern with increasing prevalence each year affecting 45% of patients admitted to the ICU and 20% of hospitalized patients (220). AKI is associated with high morbidity and mortality due to the lack of powerful biomarkers for early detection and the limitation of fully successful therapeutic strategies (221).

In the past few years, it has been shown that patients surviving an episode of AKI have a higher risk of developing CKI. CKI leads to ESRD, where the only therapies available are dialysis or transplantation (222, 223). Both modalities have their limitations and disadvantages. While dialysis is associated with a high morbidity and mortality, patients in need for renal transplantation are mostly waiting for several years before a suitable renal allograft is available. In addition, allograft recipients also need life-long immune suppression, which increases the risk for general infections and cancer (224).

### AKI induced by ischemia/reperfusion

I/R is the main cause of AKI. It is caused by a reduction of blood supply to the kidney followed by a restoration of perfusion. This decreased oxygenation produces depletion of ATP, metabolic impairment, apoptosis, production of ROS, and it is worsened during the reperfusion period, causing sterile inflammation, vasoconstriction and oxidative damage (225, 226). AKI I/R is often the result of arterial occlusion during surgery, congestive heart failure, or kidney transplantation (227). Animal models of AKI I/R have been extensively used in different species for decades. The most common procedure consists in clamping the renal artery and vein for a desired amount of time and then releasing the clamps allowing reperfusion (228).

Key to therapy is the optimal timing and dosage. Studies with BM-MSC agree that starting treatment 1 h after injury results in a better outcome, as cells are able to engraft to the kidney to a higher degree (229, 230). Interestingly, a report using human adipose stromal vascular fraction (SVF) before the induction of AKI I/R revealed an increased retention of cells in the kidney and enhanced tubular cell proliferation (152). Authors agree that the higher the dose, the worse the outcome, mostly due to cell emboli formation. The optimal cell concentration range found is 5 × 10^5^ − 1 × 10^6^ (164, 231).

AKI-I/R has a very complex pathophysiology in which several mechanisms are involved. One of the main events is apoptotic cell death of proximal tubular cells. These cells are particularly sensitive to hypoxia. An interesting study by Cantaluppi et al. demonstrated that endothelial progenitor cells (EPC)-derived MVs deliver miRNA to reprogram hypoxic proximal tubular cells into a regenerative phenotype (174). This anti-apoptotic effect mediated by miRNA transfer was also seen using hBM-MSC-MVs, in which the pre-treatment with RNAse abolished the aforementioned effect (175). Tubular cell proliferation after ischemia was also investigated in a study using hASC, in which an upregulation of SOX9 (tubular cell repair marker) was observed (153). Under physiological conditions, antioxidant enzymes present in the kidney are able to scavenge ROS. However, in AKI I/R these enzymes are overwhelmed by the massively increased production of ROS. BM-MSC are able to reduce oxidative stress by decreasing the amount of malondialdehyde, superoxide dismutase, and glutathione peroxidase in post ischemic kidney tissue (232). A similar effect was found using hUC-MSC (155). The underlying mechanism was further investigated in a study with human Wharton's jelly (WJ)-MSC-EVs in which they found that the antioxidant effect observed was the result of the Nrf2-ARE pathway activation, which regulates the expression of antioxidant proteins (159).

Another very important pathophysiological mechanism involved in AKI I/R is the so-called sterile inflammation. In this regard, ASC have shown to downregulate the expression of inflammatory cytokines and chemokines (IL-6, TNF-α, IL-1β, IFN⋎, CXCL2) and upregulate the anti-inflammatory cytokine IL-10 (233, 234). Macrophage number and migration was reduced in a study using iPSC and hWJ-MSC-EVs where the effect was observed in terms of downregulation of MCP-1 and CX3CL1 (chemoattractant factor for macrophages) (160, 235). I/R injury eventually leads to kidney fibrosis, which is the deposition of connective tissue in the renal parenchyma. Studies using hiPSC with renal lineage and amniotic fluid-derived stem cells (AFSC) revealed a decrease in interstitial fibrosis and myofibroblast formation (164, 165). In another study using hWJ-MSC the reduction of overall fibrosis was attributed to a HGF/TGF-β shift to HGF (170).

Stem cells from diverse sources and their EVs seem to be efficiently counteracting the main pathophysiological events described for AKI I/R. However, the mechanisms underlying the beneficial effects observed are still elusive. More studies focusing not only on the outcome, but also on the mechanisms of action, are necessary previous to safely introducing MSC therapy into clinical practice. It is also noteworthy that all studies evaluated in this review focused on the acute phase of the injury, neglecting the progression of AKI to CKI. For that reason, further investigations are needed to understand the long-term effects.

### AKI induced by chemotherapy

Cisplatin is a chemotherapeutic drug largely used for the treatment of several solid organ tumors and it is considered a gold standard treatment in oncology. Its nephrotoxic side effect has been widely described and reported in literature (236). Nephrotoxicity occurs in approximately 20–30% of patients treated with cisplatin due to its accumulation in proximal tubular cells where it induces apoptosis. Cisplatin has been used for decades in animal experiments in order to induce both AKI and CKI, and it represents a commonly used model to study therapeutic effects of MSC (237).

The first study involving MSC as a therapeutic approach for the treatment of cisplatin-induced AKI dates back to 2004, when Morigi et al. described the effect of allogeneic stem cell administration in a mouse model (238). In this study, BM-MSC and BM derived hematopoietic stem cells (HSC) were tested and compared, showing that only BM-MSC lead to a functional and morphological restoration of the kidney. The benefits of allogeneic MSC in cisplatin-induced AKI treatment are documented in several studies, where different cell sources, administration routes and approaches have been tested to better understand the mechanisms of action involved (55, 87, 239).

In xenogeneic studies, good results in terms of survival rate, renal function and morphology were achieved through the application of different types of hMSC, such as hBM-MSC (195, 196), hAFSC (182, 192), hESC-MP (190), hUC-MSC (32, 179, 187, 189), hUCB-MSC (31, 85). Recently, hASC have become the most widely used cell type in animal experiments, resulting in good treatment results in AKI (180, 183, 184, 186). The therapeutic effect of hASC has been demonstrated for the first time in a cisplatin induced AKI model in 2012 by Kim et al. where cells and CM were tested and both resulted in a reduction of renal tissue damage and an increase in survival rate (79). This already suggested that CM may replace cell infusion, which is of importance when considering risk/benefit ratio. Ashour et al. compared the therapeutic effect of hASC and hAFSC (181). Results show that when hAFSC were used, higher regenerative and proliferative activities were observed, compared to hASC. The authors wanted to underline that primitive MSC, such as hAFSC, have biological advantages over adult MSC and therefore should be chosen for clinical applications.

So far, the anti-apoptotic mechanism seems to be the most widely recognized in cisplatin models. Yao et al. investigated the role of apoptosis when hASC are administrated in a cisplatin-induced rat model (184). The histological analysis showed that in treated animals, the percentage of apoptotic cells was reduced and the tubular cell proliferation increased. *In vitro* and *in vivo* experiments indicated that MSC might produce specific cytokines, which are able to mediate an anti-apoptotic function through p38-MAPK, Bax, and Bcl-2 pathways. Wang et al. investigated the role of the transcriptional factor hypoxia-inducible factor subunit 1 alpha (HIF-1α) as anti-apoptotic candidate (183). HIF-1alpha has been effective in the treatment of various kidney diseases, activating downstream renal protective gene expression. Supporting this notion, genetically modified hASC overexpressing HIF-1α mediated an increased anti-apoptotic effect. The approach to genetically modify MSC has been also used in another interesting study where hESC were modified to overexpress VEGF (193). It is known that VEGF plays an important role in the renoprotective action mediated by MSC and it has been demonstrated that the VEGF-modified hESC exert a more pronounced renoprotective effect compared to the wild type ones. This results from an anti-apoptotic mechanism.

Since no cell engraftment into the kidneys was found, a possible explanation of the successful result achieved in the AKI treatment has been attributed to a possible secretion of other factors, promoted by the upregulation of VEGF, that contribute to the recovery of the kidney. High levels of VEGF were also found in a study in which the animals were treated with hUCB-MSC 1 day after induction of AKI (31). These animals showed a better outcome, which was associated with increased immunomodulatory activity and upregulation of anti-inflammatory cytokines, suggesting time dependency. Production of anti-inflammatory cytokines and anti-apoptotic proteins is also reported in another study in which a reduction of TNF-α, NF-kB, p38-MAPK, MCP-1, Bax, and caspase-3 is presented when cisplatin-induced AKI animals are treated with BM-MSC compared to the healthy counterpart (240). In animal models of cisplatin-induced AKI, MSC therapy has shown encouraging therapeutic effects, mainly involving renoprotective trophic effects. It has been largely demonstrated that the anti-apoptotic mechanism is one of the main effects mediated by MSC, even though further analyses are necessary to clarify how this is accomplished.

There is only one ongoing phase I clinical trial aiming to test the safety and efficacy of MSC to repair the kidney and improve renal function in patients with cisplatin induced AKI and solid organ cancer (200). In this study, the patients are treated with a single systemic infusion of allogeneic hBM-MSC with a 1-month follow up.

### Diabetic nephropathy

One of the most costly complications arising from long-term sustained hyperglycemia is DN, which represents the most frequent cause of ESRD (more than 35% in the U.S.) (https://www.usrds.org/2017/view/Default.aspx). The onset and progression to ESRD is mostly silent and develops slowly. Abnormal kidney function is often detected only by chance after a five to ten year duration of diabetes (241–243). The pathological changes in diabetic nephropathy are well-characterized and follow a progressive pattern, where ECM deposition in the base membrane of glomeruli and tubular tissues are the main findings (244). The molecular mechanisms have been extensively studied as result of the comprehensive research of glucose metabolism and the effects of side by-product of these pathways *in vitro* in kidney tissues (202, 245–247). The development of diabetic animal models such as the STZ model (diabetes type 1, T1D) or leptin receptor deficient models (diabetes type 2, T2D) are essential to understand the progression that might occur in different types of diabetic patients (248).

The use of MSC to ameliorate this complication is not new. In 2006 Lee et al. demonstrated that intra-cardiac (IC) infusion of hBM-MSC had a double benefit to STZ diabetic mice by reducing mesangial matrix deposition and restoring pancreatic damage (84). The restoration of insulin secreting tissue has not been confirmed in latter studies. In a similar STZ model Ezquer et al. administered BM-MSC and observed significantly reduced glomerulosclerosis and ECM deposition, in both presence and absence of glucose normalization due to pancreatic regeneration (249–251). Similar histological end-points (such as ECM and collagen deposition reduction) support the benefits of stem cell-based therapies in this setting (252–258). Regarding functional parameters, most of the studies have reported an improvement of albuminuria in severely damaged kidneys, not only in STZ models, but also in diabetes type 2 models (259, 260).

Distinctly, Nagaishi et al. repeatedly confirmed the benefits of BM-MSC in insulin resistant and insulin depleted diabetic nephropathy models with positive outcomes using not only cells, but also CM (259). This indicates that EVs and cytokine release might be more relevant for therapeutic actions than cells themselves (197). Similarly, the immunomodulatory properties are reported and resulted in the reduction of macrophage infiltration, MCP-1 expression in tissues, and reduction of inflammatory cytokines (89, 251, 259, 261, 262). These findings clearly suggest that the metabolic environment has an important impact on the performance of MSC (by deactivation or detrimental shift of their properties). Again, further *in vitro* and *in vivo* experiments are required to answer still open questions. Interestingly, none of the studies reported signs of a host derived native immune response, even though different allogeneic and xenogeneic cell types (260, 263), and different concentrations of the same were used (84, 197–199). The positive outcome observed are similar in most of the studies, where ECM deposition, urinary albumin/creatinine ratio, and less epithelial to mesenchymal transition (EMT) is observed.

There are currently two ongoing clinical trials, which study the safety, feasibility and tolerability of allogeneic hMSC-based interventions and the efficacy in DN patients (202, 203). Meanwhile, a completed clinical trial performed by Packham et al. in 2016 revealed no allogeneic adverse effects in patients treated with BM derived mesenchymal precursor cells compared to placebo (201). Nevertheless, the results regarding renal functional improvement were inconclusive, suggesting that larger populations and long-term studies might be needed to address this issue. Alloreactivity and the patients immune response are critical aspects to be assess before exploring the benefits reported in the literature. However, these topics are scarcely investigated in the field of preclinical research suggesting that further studies are needed.

### Polycystic kidney disease

PKD is a renal disease that leads to ESRD and is the fourth most common cause of chronic renal insufficiency. The pathology can arise sporadically; however, most forms are hereditary (264). Two of the best characterized forms of PKD are: autosomal dominant polycystic kidney disease (ADPKD) that occurs typically in adults and autosomal recessive polycystic kidney disease (ARPKD) that mainly affects infants and is associated with significant neonatal mortality and childhood morbidity (264, 265). The most likely mechanism is an alteration of the proteins signaling function within the cell and primary cilia. As a result, cells lining the renal tubules may grow and divide abnormally causing the growth of numerous cysts (266–268).

Nowadays, there are just a few therapies available for PKD patients. End-stage PKD patients usually undergo dialysis or renal transplantation. There is a strong need for more advanced therapeutic measures, due to poor life quality in long-term dialysis treatment, expensive insurance costs and increasing organ transplant waiting lists. Since the end of the last century, different types of PKD animal models have been characterized. In this context, the characteristic mutation in these animals has either been induced or developed spontaneously (269–272).

In the last years, two studies have been published that show the possible therapeutic effects of allogeneic stem cells in rats. Franchi et al. have shown that a single injection of BM-MSC isolated from healthy SD rats could improve the kidney function in PCK rats, partially restoring renal function (273). This study points out two of the potential effects of stem cells: the paracrine effects, by the release of cytokines, such as SDF1, VEGF, and HGF, and the ability of the cells, after injection, to acquire characteristics of endothelial cells, suggesting transdifferentiation of these cells. Kelly et al. instead, demonstrated that only repeated injections of primary renal tubular cells isolated from healthy SD ameliorate renal function (274). The authors have documented a clear engraftment of donor stem cells after transplantation, which leads to a decrease of total cyst volume and fibrosis and also improves vasculature, resulting in better delivery of oxygen and nutrients. The aforementioned studies have shown that MSC ameliorate renal function and limit the cysts formation in PKD animal model. Further studies should be performed in order to assess whether hMSC can also be safely applied in PKD.

Interestingly, despite the low numbers of preclinical evaluations, there is already one finished phase I clinical trial which had the aim to investigate the safety and efficacy of autologous hBM-MSC to improve the renal function in patients affected by ADPKD (204). Here, cultured BM-MSC were administrated to patients, with an 18-month follow up. Due to the limited number of samples, and lack of a control group, in this study it was not possible to demonstrate the efficacy of stem cell therapy. However, it demonstrated the safety and tolerability of IV infusion of autologous MSC. Currently, in literature there are various clinical research dedicated to safety and tolerability evaluation of MSC infusion in different pathological conditions. However, as indicated by the authors, several pathways are related with PKD progression, such as inflammation, cyst proliferation, and apoptosis. Since the mechanisms highlighting the positive effects are not yet fully clarified, it would be advised to do an evaluation of these observed mechanisms of action in relation to a long term MSC injection therapy.

### Kidney transplantation

KTx is the treatment of choice for patients with ESRD providing better quality of life and increasing life expectancy. However, the long term survival of the grafts is not yet optimal (275–277). This is due to organ shortage and the subsequent access to extended-criteria and brain dead donors (278–281). Moreover, the main cause of kidney transplantation failure is the development of interstitial fibrosis and tubular atrophy, which is a progressive kidney dysfunction associated also with vascular occlusion and glomerulosclerosis (282, 283). Therefore, stem cell treatment arises as a possibility to create new regimes in combination with immunosuppressive drugs or to minimize the doses needed to prevent rejection while preserving renal function (9, 284).

The main points of interest regarding stem cell therapy in kidney transplantation are their competence to modulate the immune response and to reduce interstitial fibrosis. BM-MSC are able to preserve renal function, reduce interstitial fibrosis and tubular atrophy by decreasing T cell and macrophage activation at 24 weeks after transplantation, in a Fisher to Lewis KTx model (81). However, in a KTx model using a complete MHC discordant donor-recipient combination, the use of BM-MSC worsens transplantation outcome, causing massive infiltration, thrombotic microangiopathy and increased IL-2 and IFN-γ expression (285). As expected, the time of administration plays an important role, as in a mouse model of fully mismatched MHC, administration of recipient-derived BM-MSC 2 days after KTx caused progressive graft dysfunction and graft rejection within 20 days, but MSCs injection 1 or 7 days before KTx, prolonged graft survival by migration of the cells to the spleen causing expansion of donor specific Treg (286). Another interesting study compared BM-MSC wild-type with MSC IDO knockout. The BM-MSC wild-type were able to induce allograft tolerance by a CD4+ T cell response impairment and by increasing the percentage of FOXP3+ regulatory T cells; whereas the MSC IDO knockout were not able to induce allograft tolerance, suggesting IDO is involved in MSC immunosuppressive effect (46). The use of BM-MSC in combination with the immunosuppressant cyclosporine-A has also been tested. It shows protection of graft function, but not improvement of animal survival as strongly as cyclosporine-A treatment alone, suggesting a potential interaction between this drug and MSC (287). In an allogeneic kidney transplantation rat model, BM-MSC decrease ED1+ and CD8+ cells and are able to reduce interstitial fibrosis and TGF-β1 when administered 3 and 7 days after KTx (288, 289).

Also in an allogeneic mouse model, BM-MSC-MVs increased graft survival by decreasing the percentage of MHCII+, CD80+, and CD86+ cells. Interestingly, also in the transplantation scenario, the pre-treatment of MVs with miRNA-146a inhibitor abolished the observed beneficial effect. ASC have been tested in a fully MHC disparate rat model suggesting prolonged graft survival by decreasing CD4+/ CD8+ratio. The mechanism involved would be upregulation of tumor necrosis factor-inducible gene 6 protein (TSG-6) by ASC *in vitro*. This upregulation causes the suppression of allo-reactive T cells by downregulation of CD44, leading to suppression of T cell activation and infiltration into the graft (290).

So far, four clinical trials using MSC in kidney transplantation have been completed. The common factor in all of them is the use of MSC in combination with immunosuppressive drugs to minimize the doses needed and therefore, reduce the incidence of adverse effect associated with their use. In the first one, conducted by Trivedi et al. high doses of donor-derived peripheral blood stem cells were used in pediatric recipients in combination with cyclosporine-A and prednisolone. After 18 months of observation, there was 100% graft survival, sustained renal function and low incidence of opportunistic infections (218). In the second one, conducted by Reinders et al. a double infusion of autologous BM-MSC was given to allograft recipients with subclinical rejection. An inhibition of donor-specific immunity was observed in five of the six patients treated 6 months after cell infusion (211). The third one, conducted by Tan et al. autologous MSC infusion, together standard and low doses of calcineurin inhibitors were used. An additional group received IL-2 receptor antibodies and standard doses of calcineurin inhibitors. The results showed that patients receiving MSC had lower incidence of acute rejection, decreased risk of secondary opportunistic infections and improved renal function after one year, compared to the patients infused with IL-2 receptor antibody (213). In the fourth one, by Ciancio et al. donor-derived BM-MSC were infused in living-related transplant recipients. Patients were additionally treated with alemtuzumab with steroid free maintenance. This combination was unable to induce tolerance and showed suboptimal graft survival (210). Further information about ongoing clinical trials is summarized in Table 5. The clinical trials completed give an overview of possible new therapeutic regimes. However, there are several questions to which there is no consensus, such as: most suitable timing for administration (before or after KTx) and most suitable dosage. This information is vital for an appropriate translation to clinical practice. Further research is needed to elucidate the aforementioned issues as well as to assess possible synergistic or antagonistic effects of MSC with the most commonly used immunosuppressive drugs.

**Table 5:** MSC clinical trials.

**Condition**	**ID**	**Title**	**Link**	**Status**	**Estimated Completion Date**	**References**
AKI induced by Chemotherapy	NCT01275612	*Ex-vivo* expanded mesenchymal stem cells to repair the kidney and improve function in cisplatin-induced acute renal failure in patients with solid organ cancers	https://clinicaltrials.gov/ct2/show/NCT01275612	Recruiting	March 2018	(200)
T2D-DN	NCT01843387	A Randomized, Controlled, Dose-Escalation Pilot Study to Assess the Safety and Efficacy of a Single Intravenous Infusion of Allogeneic Mesenchymal Precursor Cells (MPCs) in Subjects With Diabetic Nephropathy and Type 2 Diabetes	https://clinicaltrials.gov/show/NCT01843387	Completed	September 2015	(201)
DN	ChiCTR-ONC-17011065	The Preliminary Clinical Trial of Treating Diabetic Nephropathy by hUCT-MSC	http://www.chictr.org.cn/showproj.aspx?proj=18779	Not yet recruiting	June 2020	(202)
DKD	NCT02585622	Novel Stromal Cell Therapy for Diabetic Kidney Disease (NEPHSTROM Study)	https://clinicaltrials.gov/show/NCT02585622	Not yet recruiting	December 2020	(203)
ADPKD	NCT02166489	Evaluation the Effect of Mesenchymal MSCs Transplantation in Patients With Chronic Renal Failure Due to Autosomal Dominant Polycystic Kidney Disease	https://clinicaltrials.gov/ct2/show/NCT02166489	Completed	January 2016	(204)
KTx	NCT02490020	A Perspective Multicenter Controlled Study On Application Of Mesenchymal Stem Cell(MSC) To Prevent Rejection After Renal Transplantation By Donation After Cardiac Death	https://clinicaltrials.gov/ct2/show/NCT02490020	Enrolling by invitation	December 2018	(205)
KTx	NCT00497926	Induction of Donor Specific Tolerance in Recipients of Living Kidney Allografts by Donor FCRx Infusion	https://clinicaltrials.gov/ct2/show/NCT00497926	Active, not recruiting	March 2031	(206)
KTx	ACTRN12615000678594	Mesenchymal Stem Cells to prevent ischemia reperfusion injury in deceased donor renal transplant recipients	http://www.anzctr.org.au/ACTRN12615000678594.aspx	Recruiting	No info	(207)
KTx	NCT02409940	A Randomized Trial to Elucidate Effect of Mesenchymal Stem Cells on Immune Modulation in Living Related Kidney Transplant Patients	https://clinicaltrials.gov/ct2/show/NCT02409940	Active, not recruiting	March 2017	(208)
KTx	NCT02561767	The Efficacy and Safety of Bone Marrow-derived Mesenchymal Stem Cells in Kidney Transplantation From Chinese Donation After Citizen Death (DCD): A Multi-center Randomized Controlled Trial	https://clinicaltrials.gov/ct2/show/NCT02561767	Unknown	October 2017	(209)
KTx	NCT00183248	Using Donor Stem Cells and Alemtuzumab to Prevent Organ Rejection in Kidney Transplant Patients	https://clinicaltrials.gov/ct2/show/NCT00183248	Completed	November 2009	(210)
KTx	NCT00734396	Bone Marrow Derived Mesenchymal Stem Cells for the Treatment of Allograft Rejection After Renal Transplantation	https://clinicaltrials.gov/show/NCT00734396	Completed	December 2012	(211)
KTx	NCT01649388	Delayed Tolerance in Recipients of Living Kidney Allografts by Donor FCRx Infusion	https://clinicaltrials.gov/ct2/show/NCT01649388	Active, not recruiting	December 2030	(212)
KTx	NCT00658073	Allogeneic Bone Marrow Mesenchymal Stem Cell Transplantation in Recipients of Living Kidney Allografts	https://clinicaltrials.gov/ct2/show/NCT00658073	Completed	October 2010	(213)
KTx	ChiCTR-ONC-11001873	Effect of co-transplantation of hematopoietic stem cell transplantation and renal transplantation in long-term outcome of allograft	http://www.chictr.org.cn/showproj.aspx?proj=7675	No info	No info	(214)
KTx	EUCTR2011-001822-81-BE	Infusion of third-party mesenchymal stem cells after renal or liver transplantation: a phase I-II, open-label, clinical study	https://www.clinicaltrialsregister.eu/ctr-search/search?query=eudract_number:2011-001822-81	Ongoing	No info	(215)
KTx	NCT00659620	Mesenchymal Stem Cell Transplantation in the Treatment of Chronic Allograft Nephropathy	https://clinicaltrials.gov/show/NCT00659620	Unknown	May 2010	(216)
KTx	NCT00498160	Induction of Donor Specific Tolerance in Recipients of Live Donor Kidney Allografts by Donor Stem Cell Infusion	https://clinicaltrials.gov/ct2/show/NCT00498160	Active, not recruiting	December 2024	(217)
KTx	CN-00448212	Donor bone marrow derived stem cell infusion in thymus and periphery: an integrated approach to achieve tolerance in cadaver renal allograft recipient	http://onlinelibrary.wiley.com/o/cochrane/clcentral/articles/212/CN-00448212/frame.html	Completed		(218)

The results involving stem cells in kidney transplantation seem promising. However, due to the immense complexity of the mechanisms involved, the translation from animal models to clinical practice seems elusive. It would be interesting to see more studies using MSC in combination with the most common immunosuppressive drugs to assess possible synergic effects.

## Conclusions

MSC are one of the mostly investigated cell types for cell based therapies. Due to their heterogeneous modes of action, they are widely studied in a variety of injuries. MSC effects seem to be mediated by immunomodulation, anti-apoptotic effect, involving secretion of paracrine and endocrine factors that act toward injury amelioration. Therefore, this review aims to highlight the latest information of MSC therapy in preclinical studies and clinical trials in diverse kidney diseases.

Although, there are multiple evidences showing that there is no consensus regarding the best administration route, dosage, timing, tissue source, and specific mode of action, due to high variances within the different kidney injury models, preclinical data suggest beneficial effects in kidney injuries.

The large complexity of the mechanisms involved, make the translation from preclinical studies to clinical trials extremely challenging. In fact, it became rapidly clear that the assumed effect does not rely on local engraftment and differentiation, but rather on trophic effects. Still, the exact mode of action is not known. Varieties of mechanisms have been postulated, varying slightly with respect to the disease entities, but still, there is no clear indication about what defines clinical efficacy. Potency assays, for instance, are still lacking to predict clinical outcomes. We fully agree with the concept proposed by Ivan Martin that a reiterative cycle is required: 1. clinical indication and proposed mechanism of action, 2. specification of the product, 3. adaptation of manufacturing, and 4. assessment of clinical efficacy in well-designed and controlled clinical trials. For optimization feed-back loops have to be implemented to learn from the results, obtained in patients, in preclinical models and corresponding *in vitro* correlates (11). Some clinical field appear to have already evolved, based on this “Bench to bedside and back” approach, such as graft-versus-host disease and Crohn's related perianal fistula (291–293).

It should be noted that animal models are not fully able to mimic the human disease, this is why preclinical data must be taken cautiously. Autologous MSC have already been used in clinical trials and are known to have a high safety and tolerability. In our search we found several clinical trials where allogeneic MSC are used. However, there is not yet a clear understanding of how MSC act in such environment. According to the stated paracrine effect of MSC, the new trend is to test MSC EVs or CM instead of their cellular counterpart. Based on preclinical studies they have been proven efficient in ameliorating kidney diseases. Despite this knowledge, further elucidation is needed for translation to clinical trials.

The use of MSC for therapeutic purposes show encouraging results, nevertheless, the complexity of these kidney diseases, together with the animal model limitations require careful investigation. We suggest that due to our current limitations in understanding cell behavior *in vivo*, there is a need for novel imaging techniques (such as OTC) to open a new horizon in imaging MSC therapeutic field.

## Author contributions

AT and CD manuscript writing, assembling, reviewing, editing. CG, SM, DP, DN, and CB manuscript writing. BY and NG manuscript editing and final approval. KB manuscript writing, editing and final approval.

### Conflict of interest statement

The authors declare that the research was conducted in the absence of any commercial or financial relationships that could be construed as a potential conflict of interest.

## Abbreviations

ADPKD: Autosomal Dominant Polycystic Kidney Disease
AFSC: Amniotic fluid-derived stem cells
AKI I/R: AKI induced by ischemia/reperfusion
AKI: Acute kidney injury
ARE: Antioxidant response element
ARPKD: Autosomal Recessive Polycystic Kidney Disease
ASC: Adipose-derived MSCs
ATG: Antithymocyte globulin
BM: Bone marrow
BUN: Blood urea nitrogen
CKI: Chronic kidney injury
CM: Conditioned medium
COX2: Cyclooxigenase-2
DiD, 1, 1-Dioctadecyl-3, 3, 3: 3-tetramethylindodicarbocyanine
DN: Diabetic nephropathy
ECM: Extra cellular matrix
EMT: Epithelial–mesenchymal transition
EPC: Endothelial progenitor cells
ESC: Embryonic stem cells
ESC-MP: Mesenchymal-like progenitors derived from embryonic MSC
ESRD: End-Stage Renal Disease
EVs: Extracellular vesicles
GM-CSF: Granulocyte-macrophage colony-stimulating factor
HGF: Hepatocyte growth factor
HIF-1α: Hypoxia-inducible factor subunit 1α
HLA: Human leukocyte antigen
HSC: Hematopoietic stem cells
IA: Intra-arterial
IC: Intra-cardiac
ICG: Indcyanine Green
IDO, Indoleamine 2: 3-dioxygenase
IGF: Insulin-like growth factor
IP: Intra-peritoneal
iPSC: Induced-pluripotent stem cells
IR: Intra-renal
IV: Intra-venous
KDC: Kidney-derived cells
KTx: Kidney transplantation
luc/neo-MSC: luciferase/neomycyn phosphotransferase-marked MSC
MCP-1: Monocyte chemoattractant protein-1
MMPs: Metalloproteinases
MRI: Magnetic resonance imaging
MVs: Microvesicles
OI: Optical imaging
PKD: Polycystic Kidney Disease
RPC: Renal progenitor cells
sCr: Serum creatinine
sNGAL: Serum Neutrophil gelatinase-associated lipocalin
STZ: Streptozotocin
SVF: Stromal vascular fraction
T1D: Type 1 diabetes
T2D: Type 2 diabetes
TIMPs: tissue inhibitors of metalloproteinases
TNF-α: Tumor necrosis factor Alpha
Treg: Regulatory T cells
U-ACR: Urine albumin to creatinine ratio
UC: Umbilical cord
UCB: Umbilical cord blood
UDSC: Urine derived stromal cells
USSC: Unrestricted somatic stem cells
WJ: Wharton's jelly
α-SMA: Alpha smooth muscle.
